# Microbiota diversity and hygienic behavior in a honey bee breeding population: Insights into *Varroa* resistance

**DOI:** 10.1371/journal.pone.0346605

**Published:** 2026-04-09

**Authors:** Maria Grazia De Iorio, Francesco Tiezzi, Giulietta Minozzi

**Affiliations:** 1 Department of Veterinary Medicine and Animal Sciences, University of Milan, Lodi, Italy; 2 Department of Agriculture, Food, Environment and Forestry, University of Florence, Florence, Italy; University of Alberta, CANADA

## Abstract

Hygienic behavior is a key trait in the western honey bee (*Apis mellifera*) associated with resistance to diseases and tolerance to the parasitic mite *Varroa destructor*. Worker bees expressing this behavior are able to detect and remove unhealthy or infested brood, thereby limiting pathogen transmission and mite reproduction. In recent years, the gut microbiota has emerged as an important factor in honey bee health, influencing immunity and disease resistance. Hygienic behavior is a form of social immune defense; however, whether gut microbiota composition contributes to variation in this disease-resistance mechanism remains poorly understood. This study investigated the relationship between gut microbiota composition and hygienic behavior in a honey bee breeding population selected for docility, honey production, and hygiene since 2015. Seventy-seven colonies were sampled at three time points between June and October 2021 for gut microbiota analysis using high-throughput sequencing. Hygienic behavior was evaluated using the average pin test score, calculated from tests performed in March and July. Significant association were identified between gut microbiota diversity and hygienic behavior, with marked seasonal pattern. Colonies with higher pin test scores showed increased alpha diversity in October, while beta-diversity analyses indicated significant difference in July. In particular, LAB genera such as *Lactobacillus, Bifidobacterium*, and *Bombilactobacillus*, were more abundant in highly hygienic colonies in July, suggesting a microbiota configurations potentially linked enhanced immune capacity and resilience to brood diseases and *Varroa* infestations. Overall, these findings support a seasonal association between gut microbiota composition and hygienic behavior in honey bees and highlight specific microbial taxa that may contribute to colony robustness. This study provides a basis for future research exploring the functional role of microbiota in social immunity and its potential integration into selective breeding strategies aimed at improving colony health.

## Introduction

Honeybees (*Apis mellifera*) are pivotal pollinators critical to both natural ecosystems and agricultural productivity worldwide. They play a fundamental role in pollinating a vast array of crops, thereby ensuring food security and biodiversity conservation [1]. However, honeybee populations face numerous challenges, among which the *Varroa destructor* mite stands out as one of the most significant threats [2]. *Varroa destructor*, originally from Asia, is an ectoparasite of *Apis cerana* with which it has co-evolved. It has become one of the most damaging pests affecting honeybee colonies globally after shifting hosts to *A. mellifera* [3]. These parasitic mites weaken bees by feeding primarily on fat body tissue and by transmitting harmful viruses that compromise their immune systems [4–6]. Infestations lead to reduced colony strength, diminished honey production, and heightened susceptibility to other stressors, ultimately contributing to colony losses [7].

Efforts to mitigate *V. destructor* infestations involve the implementation of different management strategies, including selective breeding programs aimed at enhancing honeybee resistance traits. Among the phenotypes targeted in selective breeding approaches, hygienic behavior represents a key resistance mechanism already widely used in apicultural selection programs, as it plays a crucial role in limiting the impact of *Varroa* infestations and reducing the spread of diseases within the colony [8]. Hygienic behavior is characterized by the ability of worker bees to detect and remove dead or infected brood from sealed cells. The enhancement of this process disrupts the reproductive cycle of *Varroa* mites and reduces the prevalence of pathogens, thereby enhancing colony health [9]. The quantification of hygienic behavior typically involves measuring the rate at which worker bees remove dead brood within a defined time interval (e.g., 24 hours), using methods such as the pin-killed brood assay, where brood is mechanically killed with a needle, or the freeze-killed brood (FKB) assay, which employs liquid nitrogen to kill the brood [10].

In addition to genetic factors, the gut microbiota of honeybees has emerged as a crucial determinant of their health and resilience [11]. The honeybee gut harbors a conserved core microbiota that plays essential roles in nutrition, immunity, and protection against pathogens [11–14]. Moreover, recent studies in Swedish honeybee population have reported differences in specific bacterial taxa between *Varroa*-surviving and *Varroa*-susceptible colonies [15,16].

Dosch et al. [17] further demonstrated that honeybee survival against deformed wing virus, transmitted by *Varroa*, was significantly enhanced with a normal gut microbiota compared to dysbiotic bees. These findings highlight the importance of microbiota composition in resistance to *Varroa* and its transmitted viruses.

Hygienic behavior is a key component of social immunity in honey bees, representing a collective defense mechanism that reduces pathogen transmission and parasite reproduction [18]. Beyond the established role of the gut microbiota in individual immunity and disease resistance [11], recent evidence suggests that gut microbiota composition may also be associated with hygienic behavior [19]. Specifically, this study reported significant differences in gut microbiome alpha diversity and in the relative abundance of specific taxa [19].

Therefore, understanding the genetic, physiological, and microbial mechanisms underlying resistance to *Varroa* is critical to developing effective strategies to safeguard bee health and ensure sustainable pollination services. In this context, this study investigates whether and how gut microbiota composition is associated with variation in hygienic behavior, analyzing colonies at different timepoints and seasons to assess the persistence of such association across sampling periods.

## Materials and methods

### Honey bee sampling and hygienic behavior assessment

During 2021, samples for microbiota analysis were collected from 77 colonies at three seasonal timepoints (June, July, and October). Due to queen loss and other colony-level disruptions occurring over the sampling period, as well as occasional data loss, not all colonies were available at each timepoint. As a result, a total of 190 samples were collected. In addition, two colonies sampled at T1 were excluded from analyses involving hygienic behavior because queen loss occurred before the second pin test, preventing the calculation of the averaged hygienic score (AvePin). Consequently, 188 samples were retained for the present analyses, distributed across timepoints as follows: 73 colonies in June (T1), 69 in July (T2), and 46 in October (T3).

For each sampling, ten worker bees per hive were randomly collected from brood frames and stored at −80° C in individual sterile Falcon tubes. All colonies belonged to a breeding population selectively bred since 2015 for docility, honey production, and hygienic behavior, and were maintained in a single apiary located in Cremella (LC), Lombardy, Italy, within a predominantly rural and agricultural landscape. All colonies therefore experienced identical local climatic conditions; based on publicly available meteorological data, mean air temperatures were approximately 15 °C during June–July and 8 °C in October 2021 (https://meteostat.net/it/). At the time of each sampling, colonies were visually inspected according to standard beekeeping practices, and no clinical signs of disease were observed.

Hygienic behavior was assessed using the pin test, which quantifies the percentage of brood cells cleaned 24 hours after mechanically damaging 50 capped cells with a needle [20]. The test was repeated twice, once in late March and once in early July, and the final hygienic score (AvePin) was calculated as the average of the two measurements, following the standard protocol [21]. Hygienic behavior was therefore assessed at the colony level, and individual sampled bees were not behaviorally characterized. Descriptive statistics for the AvePin scores are reported in S1 Table.

### DNA extraction and next generation sequencing

For each colony, the guts were dissected from 10 worker bees under sterile conditions, by removing the digestive tract from the sting area under a laminar flow hood. The guts from the ten individuals were then pooled together, and DNA extraction was performed on each pool using the Blood & Tissue Genomic DNA Extraction kit (Fisher Molecular Biology, Waltham, MA, USA), following the manufacturer’s protocol. Work surfaces and all instruments were carefully sterilized between colonies to prevent cross-contamination. The V3–V4 region of the 16S rRNA gene was amplified following the Illumina 16S Metagenomic Sequencing Library Preparation protocol using standard primers and sequenced using the Illumina NovaSeq platform, producing paired-end 250-bp reads. Further details can be found in De Iorio et al. [22,23].

### Bioinformatic analysis

The 190 FASTQ files obtained from sequencing were subsequently processed in R (version 4.3.1; R Core Team, 2023). Initially, sequence processing and denoising were performed using the Divisive Amplicon Denoising Algorithm 2 (DADA2) pipeline, with customized truncation parameters of 245 bp (forward) and 240 bp (reverse) applied via the “filterAndTrim” function [24]. Taxonomic assignment was performed using the SILVA reference database (v132) using a minimum confidence threshold of 2.

To minimize sequencing noise and focus on bacteria with greater ecological significance, only amplicon sequence variants (ASVs) present in at least 50% of samples were retained [25,26]. Normalization was achieved through rarefaction, standardizing each sample to a total of 10,000 reads for comparative analysis. The representativeness of bacterial communities within the rarefied core microbiome was assessed using rarefaction curves generated with the Vegan package [27].

To characterize within-sample microbial richness and evenness in the gut microbiota community, alpha diversity indices (Chao1, Shannon, Simpson, abundance-based coverage estimator (ACE), and Fisher) were calculated using the Phyloseq and Vegan packages [27,28] and z-score normalization was applied via the “scale” function in R. Additionally, to evaluate differences in microbial community composition among hygiene groups, β-diversity was assessed using Bray-Curtis dissimilarities with the Phyloseq package in R, to evaluate differences in microbial community structure among hygiene groups.

### Statistical analysis

To investigate the relationship between microbial composition and hygienic behavior, we analysed the average pin test score, obtained as the mean score resulting from the tests conducted in March and July. According to previous studies, colonies that removed more than 90% of pin-killed brood within 24 h were classified as highly hygienic [29–31]. In order to explore intermediate levels of hygienic behaviour and facilitate interpretation, the scores were grouped as in Gebremedhn et al. [31] into three categories: LowHy (< 0.53), MedHy (0.53–0.90), and HiHy (> 0.90).

In addition to this three-level classification, dichotomous contrasts were performed by comparing HiHy against all others and LowHy against all others.

All microbiota analyses were performed separately for each sampling timepoint (June, July, and October), and samples from different timepoints were not combined within the same statistical models. All analyses were conducted for both the categorical variables (three groups) and the dichotomous contrasts (two groups). The association between α-diversity indexes and pin test groups was evaluated at each timepoint using the “kruskal.test” function in R, and when significant, a post-hoc analysis was conducted using the “dunn.test” function. Additionally, linear regression analysis was performed at each timepoint to assess the relationship between AvePin scores as continuous variable and α-diversity indexes, using the “lm” function.

To further account for repeated sampling of the same colonies across timepoints, additional analyses were conducted using the full dataset including all available samples (n = 188). In this case, α-diversity indices were analysed using linear mixed-effects models, including hygienic behaviour (either as the categorical variable or as the continuous variable), genetic line (GL), and sampling timepoint as fixed effects, as well as the interaction between hygienic behaviour and timepoint. Colony identity was included as a random effect to account for repeated measurements of the same colonies across timepoints. These models were implemented using the “lme” function from the nlme package in R.

Differences in β-diversity between groups were assessed using PERMANOVA, performed with the “adonis2” function in the Vegan package [27], with Bray-Curtis distance as the dependent variable and scores as independent variables.

In addition to the timepoint-specific analyses, β-diversity was also analysed on the combined dataset including all samples with available metadata (n = 188). In these analyses, PERMANOVA models included AvePin groups, genetic line (GL), and sampling timepoint as fixed effects, as well as the interaction between AvePin and timepoint. To account for repeated sampling of the same colonies, permutations were constrained within colony identity using the “strata” argument in the adonis2 function.

Partial Least Squares Discriminant Analysis (PLS-DA) was also performed at all timepoints to evaluate the specificity, sensitivity, and accuracy of pin test group categorizations based on the microbiota, using the “plsda” function with Mdatools package [32]. Moreover, differential abundance analyses were performed using the DESeq2 package at all timepoints to identify if and which ASVs varied between pin test groups, and results were visualized with volcano plots. Because rarefaction can bias abundance-based comparisons by altering absolute read counts, DESeq2 analyses were conducted on the non-rarefied dataset to avoid artefacts introduced by rarefaction-based normalization [26].

## Results

### Alpha diversity

Significant associations between alpha diversity and hygienic behavior were detected, although these effects were time-dependent.

**Fig 1:**
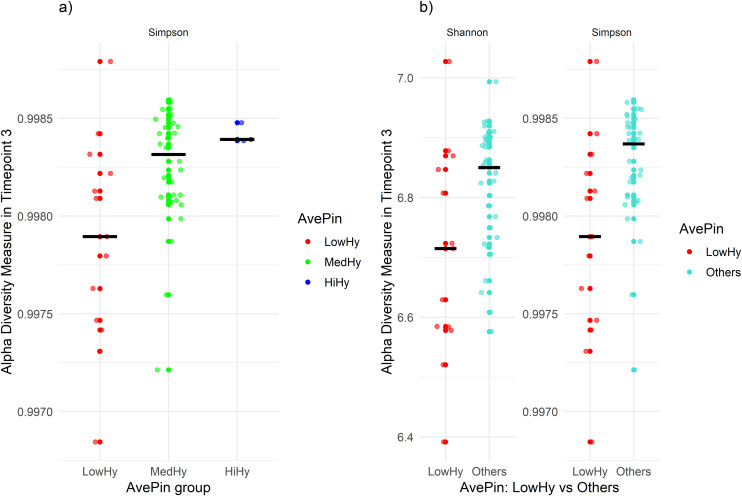
Alpha diversity indexes at timepoint 3 in relation to AvePin. a) Comparison across hygiene groups: LowHy, MedHy, and HiHy. b) comparison between the less hygienic group (LowHy) and the others. Black bars represent the median.

At timepoint 3 (October), a significant difference among hygiene groups was identified using the Simpson index, with *p*-values of 0.03, as detailed in Table 1. The post-hoc Dunn tests showed that this difference was driven by the comparison between the low and medium groups (Table 2), with higher levels of α-diversity observed in the medium group (Fig 1).

**Table 1:** Kruskal-Wallis results for alpha diversity indexes by AvePin groups at each timepoint.

Trait	Timepoint	Index	χ²	Df	*P*-value
AvePin	T1	Fisher	1.28	2	0.53
		Shannon	0.56	2	0.75
		Simpson	0.84	2	0.66
		Chao1	1.55	2	0.46
		ACE	0.93	2	0.63
		Observed	1.28	2	0.53
	T2	Fisher	1.84	2	0.40
		Shannon	0.35	2	0.84
		Simpson	0.41	2	0.81
		Chao1	3.58	2	0.17
		ACE	2.48	2	0.29
		Observed	1.84	2	0.40
	T3	Fisher	2.34	2	0.31
		Shannon	5.72	2	0.06
		Simpson	7.33	2	0.03*
		Chao1	3.58	2	0.17
		ACE	2.85	2	0.24
		Observed	2.34	2	0.31

Df = degrees of freedom.

**Table 2:** Post-hoc Dunn test results for alpha diversity indexes by pin test traits.

Trait	Timepoint	Index	Comparison	Z Test	*P*-value
AvePin	T3	Simpson	LowHy vs HiHy	1.98	0.07
			MedHy vs HiHy	0.76	0.67
			MedHy vs LowHy	−2.44	0.02*

When using the dichotomous contrast LowHy vs. Others, additional significant differences emerged at the same timepoint for both Shannon (*p* = 0.02) and Simpson (*p* = 0.01) indices (S2 Table).

Moreover, the regression analysis conducted using AvePin as a continuous variable supported time-specific association between microbial diversity and hygienic behavior. At timepoint 2 (July) significant association were detected for Fisher, Chao1, Ace, and Observed ASV, while at timepoint 3, significant effects were observed for Shannon and Simpson (Table 3).

**Table 3:** Regression results for alpha diversity indexes and average pin test scores.

Timepoint	Index	Estimate	Std. Err	T-value	*P*-value
T1	Fisher	−0.78	0.57	−1.37	0.18
	Shannon	−0.78	0.57	−1.36	0.18
	Simpson	−0.72	0.57	−1.26	0.21
	Chao1	−0.79	0.57	−1.39	0.17
	ACE	−0.59	0.57	−1.04	0.30
	Observed	−0.79	0.57	−1.39	0.17
T2	Fisher	−1.14	0.57	−2.01	0.05*
	Shannon	−0.38	0.58	−0.66	0.51
	Simpson	0.10	0.59	0.18	0.86
	Chao1	−1.25	0.57	−2.21	0.03*
	ACE	−1.17	0.57	−2.07	0.04*
	Observed	−1.12	0.57	−1.96	0.05*
T3	Fisher	1.16	0.69	1.69	0.10
	Shannon	1.53	0.67	2.28	0.03*
	Simpson	1.68	0.66	2.54	0.01**
	Chao1	1.13	0.69	1.64	0.11
	ACE	1.18	0.69	1.73	0.09
	Observed	1.17	0.69	1.70	0.10

Std. Err = Standard error.

Taken together, these results indicate that the association between alpha diversity and hygienic behavior emerges primarily at the end of the season, with colonies exhibiting lower hygiene consistently showing reduced microbial diversity, particularly in October, while no significant differences were observed at timepoint 1 (June) across any analysis performed.

Because some colonies were sampled at multiple timepoints, additional analyses were performed on the combined dataset including all samples using linear mixed-effects models to account for repeated sampling of the same colonies.

When AvePin was included as a categorical variable, no significant main effects of AvePin groups were detected for any alpha diversity index. However, significant interactions between AvePin and timepoint were observed for the Simpson and Chao1 indices (S3 Table).

When hygienic behaviour was analysed as a continuous variable, the main effect of AvePin remained non-significant, whereas the interaction between AvePin and timepoint was significant for all alpha diversity indices (Table 4).

**Table 4:** Results of linear mixed-effects models testing the association between alpha diversity indices and AvePin scores across all samples. The table reports the F-values and *p*-values for each fixed effect included in the model (AvePin, genetic line, sampling timepoint, and the interaction between AvePin and timepoint.

Index	Effect	F-value	*P*-value
Shannon	AvePin	0.02	0.88
	Genetic line	1.73	0.12
	Timepoint	10.79	5.31E-05***
	AvePin:Timepoint	3.43	0.04*
Fisher	AvePin	0.88	0.35
	Genetic line	0.43	0.88
	Timepoint	7.38	9.86E-04***
	AvePin:Timepoint	3.67	0.03*
Simpson	AvePin	0.94	0.33
	Genetic line	2.19	0.05
	Timepoint	16.22	6.84E-07***
	AvePin:Timepoint	4.31	0.02*
Chao1	AvePin	1.31	0.26
	Genetic line	0.16	0.99
	Timepoint	13.21	7.29E-06***
	AvePin:Timepoint	4.95	0.01**
ACE	AvePin	0.72	0.40
	Genetic line	0.21	0.98
	Timepoint	15.27	1.43E-06***
	AvePin:Timepoint	4.31	0.02*
Observed	AvePin	0.87	0.35
	Genetic line	0.44	0.88
	Timepoint	7.20	1.15E-03***
	AvePin:Timepoint	3.71	0.03*

### Beta diversity

Differences in microbiota β-diversity among AvePin groups were explored with PERMANOVA and PLS-DA analyses.

PERMANOVA identified a significant association at timepoint 2 when comparing HiHy colonies to the others (*p* = 0.04; Table 5), indicating differences in microbial community composition between highly hygienic colonies and the rest of the population during mid-season. Similarly, PLS-DA classification showed that the high group was better distinguished from the others, particularly at timepoint 2, where it exhibited higher specificity (1) and accuracy (0.913) (S4 Table). Across all timepoints, classification accuracy for the HiHy vs. Others contrast was systematically higher than that of LowHy vs. Others, suggesting that distinctions in microbial community structure are more pronounced for highly hygienic colonies than for poorly hygienic ones (S4 Table).

**Table 5:** PERMANOVA results for microbiome composition by pin test groups.

Trait	Timepoint	Df	SumSqs	R2	F-value	*P*-value
AvePin	T1	2	0.19	0.03	1.03	0.38
	T2	2	0.23	0.04	1.35	0.08
	T3	2	0.15	0.05	1.17	0.16
AvePin: HiHy vs other	T1	1	0.12	0.02	1.27	0.14
	T2	1	0.14	0.02	1.6	0.04*
	T3	1	0.07	0.02	1.07	0.34
AvePin: LowHy vs other	T1	1	0.09	0.01	0.92	0.57
	T2	1	0.1	0.02	1.2	0.22
	T3	1	0.08	0.03	1.3	0.12

Df = degrees of freedom.

To account for repeated sampling of the same colonies across timepoints, an additional PERMANOVA analysis was performed on the combined dataset including all samples (n = 188), constraining permutations by colony identity. In this model, significant effects were detected for AvePin groups, genetic line, and timepoint, and the interaction between AvePin and timepoint was also significant (Table 6). Among these factors, timepoint explained the largest proportion of variance in microbiome composition, whereas the interaction between AvePin and timepoint accounted for about 3% of the variance, while AvePin groups alone explained approximately 1%.

**Table 6:** PERMANOVA results testing the effects of pin test groups (AvePin), genetic line, and timepoint on microbiome composition across all samples (n = 188).

Trait	Df	SumSqs	R2	F-value	*P*-value
AvePin	2	0.16	0.01	0.95	3.00E-03**
Genetic line	7	0.58	0.03	0.98	1.00E-03***
Timepoint	2	1.06	0.06	6.20	1.00E-03***
AvePin:Timepoint	4	0.45	0.03	1.31	0.05*

Df = degrees of freedom.

Moreover, Differential Abundance Analysis was conducted using the non-rarefied datasets to identify changes in the abundance of Amplicon Sequence Variants (ASVs) across pin test groups across all timepoint.

**Fig 2:**
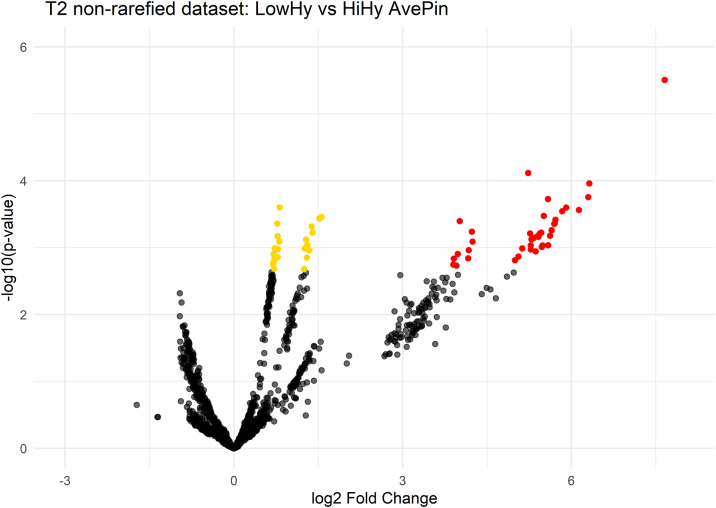
Volcano plots of ASVs differing between LowHy and HiHy (AvePin) at T2, non-rarefied data. ASVs with adjusted *p*-values smaller than 0.05 are illustrated in yellow, while ASVs with adjusted *p*-values smaller than 0.05 and log2 fold change smaller than −2 or log2 fold change larger than 2 are shown in red.

Consistently with PERMANOVA results, significant differences were observed only at timepoint 2, where 64 ASVs were more abundant in HiHy colonies compared to LowHy colonies (padj < 0.05). Among these, 40 ASV belonged to *Lactobacillus* genera (log2FC > 2), 14 to *Bifidobacterium*, and 10 to *Bombilactobacillus* (Fig 2). Notably, no ASVs were found to be significantly depleted in HiHy colonies under the applied significance and effect size thresholds, indicating a directional pattern of enrichment rather than reciprocal gains and losses across groups.

## Discussion

The pin test is a widely used method for assessing hygienic behavior in honeybee colonies, providing a standardized measure of their ability to detect and remove compromised brood. While this trait is generally associated with disease resistance, its role in *V. destructor* tolerance is particularly relevant [21,33]. Hygienic colonies can limit mite reproduction and population growth by promptly removing infested brood, thereby reducing the overall parasite load and mitigating its harmful effects on colony health [8,9].

Hygienic behavior in honey bees is influenced by several environmental factors, including seasonal changes, climatic conditions, and nectar flow availability, which can affect colony response dynamics [34]. Given this variability, standard guidelines recommend conducting the pin test at least twice a year and averaging the results to obtain a more reliable measure of a colony’s hygienic capacity [21]. Accordingly, in this study the pin test was carried out twice (March and July), and the average score (AvePin) was used as a colony-level phenotype in subsequent analyses.

Previous research on both *A. mellifera* and *A. cerana* suggests an association between microbiota and resistance to pathogens, including *Varroa*-transmitted viruses [15–17,35,36]. Given that hygienic behavior represents a key component of *Varroa* tolerance, exploring its association with microbial composition provides relevant context within the broader framework of disease resistance. Moreover, a recent study showed that worker bees actively performing hygienic behavior differ in gut microbiota composition and alpha diversity compared to non-performing workers within the same colonies [19].

All colonies analyzed in the present study belonged to a structured breeding population and were therefore genetically related, being subdivided into eight distinct genetic lines based on shared ancestry. For this reason, it is important to consider the role of host genetic background when interpreting the results. In fact, host genetic effects within this dataset have been previously evaluated and discussed in detail in a separate study [22]. Importantly, in the present study colonies classified into the low and medium hygienic behavior groups were heterogeneous and included representatives from all eight genetic lines, indicating that these categories were not driven by lineage-specific effects. The high hygienic behavior group, although numerically smaller, comprised colonies belonging to four different genetic lines (A, E, G, and H). Notably, two of the lines that were previously reported to differ most strongly in gut microbiota composition within this breeding population, namely A and H [22], were both represented within the high hygienic group. Taken together, these observations indicate that the differences observed among hygienic behavior groups cannot be readily attributed to genetic lineage alone, and therefore provide a suitable framework to further explore how hygienic behavior itself relates to microbiota composition and colony-level traits.

The results on alpha diversity indicate that microbiota composition is linked to hygienic behavior, although in a complex and context-dependent manner. Colonies with higher AvePin values showed increased alpha diversity in October, suggesting that enhanced microbial diversity later in the season may contribute to improved colony resilience and an increased ability to counter pathogen pressure following peak *Varroa* infestation. Similarly, individual-level observations have shown that hygiene-performing workers exhibit higher gut microbiota alpha diversity compared to non-performing workers [19]. Additional mixed-effects analyses conducted on the combined dataset confirmed that the association between hygienic behaviour and alpha diversity is time-dependent, as indicated by the significant interaction between hygienic behaviour and sampling timepoint across multiple diversity indices (Table 4).

Regression analysis supported this pattern, revealing significant associations between AvePin and multiple alpha diversity indices in both July and October. At timepoint 2, these associations were negative (Fisher, Chao1, ACE and Observed), whereas at timepoint 3 they became positive for Shannon and Simpson, indicating a seasonal shift in the direction of the relationship (Table 3). This suggests that changes in microbial diversity may reflect different hygienic strategies across the season, with reduced diversity potentially representing a rapid and targeted response under summer stress conditions, while increased diversity in autumn may support long-term colony stability in preparation for overwintering. Similarly, Marche et al. [36] reported significant microbiota differences in *Varroa*-infested colonies in October, correlating these changes with *Deformed Wing Virus* loads. Moreover, slight differences in alpha diversity were reported also by Thaduri et al. [15] between *Varroa*-resistant and *Varroa*-susceptible colonies always in October. Together, these findings suggest that the most pronounced microbiota differences were observed at timepoint 3 (October), a critical period following peak *Varroa* infestation. This highlights the key role of hygienic behavior in shaping colony health at the end of the season, influencing how well colonies prepare for overwintering.

Beta diversity analyses further support the hypothesis that microbiota composition in July differs among colonies with varying levels of hygienic behavior, suggesting that microbial community structure in highly hygienic colonies may be more resilient to external perturbations. Consistently, when all samples were analysed together while accounting for repeated sampling of colonies, PERMANOVA results showed that timepoint represented the main driver of microbiota variation, while hygienic behaviour explained a smaller but significant fraction of the observed variance, particularly through its interaction with timepoint (Table 6). Dosch et al. [17] similarly found that a stable microbiota composition enhances viral tolerance of Deformed Wing Virus in *A. mellifera*. Furthermore, Svobodová et al. [16] highlighted that even if microbiota composition appears similar between *Varroa*-resistant and -susceptible honey bees, specific strain-level differences exist.

Differential abundance analysis showed that the differences observed in July were driven mainly by taxa belonged to *Lactobacillus,* with additional contributions from *Bifidobacterium*, and *Bombilactobacillus* that were significantly more abundant in highly hygienic colonies compared to less hygienic ones. This pattern is consistent with previous studies showing that lactic acid bacteria (LAB), in particular *Bifidobacterium* and *Lactobacillus*, play key roles in honey bee immunity and disease resistance, and have been previously linked to *Varroa* infestations [16,17,36–40]. Notably, Tola et al. [19] similarly identified *Apilactobacillus kunkeei* as more abundant in hygiene-performing workers, further supporting the biological relevance of LAB in the context of hygienic behavior. Although the present study does not investigate causal mechanisms, the observed association between LAB abundance and hygienic behavior may be biologically meaningful, as hygienic removal of infested brood directly impacts *Varroa* reproductive success.

It is important to consider that differences in results between timepoints may also be attributed to the natural seasonal variations in microbiota structure. Many studies, including analyses conducted on this dataset, have provided evidence that the most influential factor in bees microbiota composition is seasonality [14,15,23,41–44]. However, because all colonies were sampled simultaneously at each timepoint, seasonal dynamics were shared across hygienic groups. While colonies may still have responded differently to seasonal or environmental stressors, potentially influencing both microbiota composition and hygienic behavior, seasonality alone is unlikely to fully account for the differences observed between hygiene categories within the same sampling date. Moreover, other investigations on this dataset reported association between microbiota composition, and honey production, further supporting the multifactorial nature of microbiota dynamics [22,23].

Taken together, these findings demonstrate that different studies highlight a connection between microbiota and honeybee health, although specific patterns vary due to the complexity of microbiota interactions with environmental conditions, pathogenic pressure, and colony traits. The present findings confirm the existence of a microbiota-hygiene link while highlighting its complexity, and its seasonal dependence. As suggested by Svobodová et al. [16], resilience to viral infections in *Varroa* surviving honey bees is likely linked to the specific properties of gut microbiota, which may have co-evolved with *Varroa* resistant traits.

## Conclusion

This study provides the first evidence linking microbiota diversity with the pin test as a measure of hygienic behavior and *Varroa* resistance in *A. mellifera*. By integrating microbiota analysis with behavioral phenotypes, this research contributes to a better understanding of how microbial communities may support colony health and resilience. The results underscore the complexity of microbiota-hygienic behavior interactions, demonstrating a strong seasonal dependence and suggesting that microbial community structure may respond differently to hygienic performance across the year. These findings highlight the potential functional relevance of specific bacterial taxa, particularly lactic acid bacteria, whose increased abundance was associated with higher levels of hygienic behavior.

Further research is needed to clarify the causal relationships between microbiota composition, hygienic traits, and disease resistance mechanisms, allowing for the development of microbiome-based strategies to improve honey bee health and colony sustainability.

## Supporting information

S1 Table Descriptive statistics for the average pin test scores at each timepoint. The table reports the minimum (min), mean, maximum (max), and standard deviation (SD) for each time point (T1, T2, T3).
(DOCX)

S2 Table Kruskal-Wallis results for α-diversity indexes at each timepoint, using dichotomous variables. The table reports the chi-squared statistic (chi²), degrees of freedom (df), and *P*-values for each α-diversity index across different timepoints. Significant results are indicated with * (*p*-value < 0.05) and ** (*p*-value < 0.01).
(DOCX)

S3 Table Results of linear mixed-effects models testing the association between alpha diversity indices and hygienic behavior group (AvePin) across all samples. The table reports the F-values and *p*-values for each fixed effect included in the model (AvePin, genetic line, sampling timepoint, and the interaction between AvePin and timepoint).
(DOCX)

S4 Table PLS-DA results based on the three-group AvePin classification and the dichotomous contrast variables. The table reports specificity, sensitivity, and accuracy for each group (low, medium, and high) at different timepoints.
(DOCX)
