# Supplementary material for: Microbiota diversity and hygienic behavior in a honey bee breeding population: Insights into Varroa resistance
Source: PLoS One. 2026 Apr 9;21(4):e0346605. doi: 10.1371/journal.pone.0346605 (PMC13065041; doi:10.1371/journal.pone.0346605)
Supplement: S1 Table — The table reports the minimum (min), mean, maximum (max), and standard deviation (SD) for each time point (T1, T2, T3). (DOCX) [file pone.0346605.s001.docx]

**S1 Table.** **Descriptive statistics for the average pin test scores at each timepoint.** The table reports the minimum (min), mean, maximum (max), and standard deviation (SD) for each time point (T1, T2, T3).

| **Phenotype** | **Timepoint** | **Min** | **Mean** | **Max** | **SD** |
| --- | --- | --- | --- | --- | --- |
| AvePin | t1 | 0.1 | 0.64 | 0.98 | 0.21 |
|  | t2 | 0.1 | 0.63 | 0.98 | 0.21 |
|  | t3 | 0.1 | 0.62 | 0.95 | 0.21 |
