# Supplementary material for: Microbiota diversity and hygienic behavior in a honey bee breeding population: Insights into Varroa resistance
Source: PLoS One. 2026 Apr 9;21(4):e0346605. doi: 10.1371/journal.pone.0346605 (PMC13065041; doi:10.1371/journal.pone.0346605)
Supplement: S2 Table — The table reports the chi-squared statistic (chi²), degrees of freedom (df), and P-values for each α-diversity index across different timepoints. Significant results are indicated with * (p-value < 0.05) and ** (p-value < 0.01). (DOCX) [file pone.0346605.s002.docx]

**S2 Table. Kruskal-Wallis results for α-diversity indexes at each timepoint, using dichotomous variables.** The table reports the chi-squared statistic (chi²), degrees of freedom (df), and *p*-values for each α-diversity index across different timepoints. Significant results are indicated with * (*p*-value < 0.05) and ** (*p*-value < 0.01).

| **Dichotomous variable** | **Timepoint** | **Index** | **chi2** | **df** | ***P*-value** |
| --- | --- | --- | --- | --- | --- |
| HiHy vs other | T1 | Fisher | 1.04 | 1 | 0.31 |
|  |  | Shannon | 0.53 | 1 | 0.47 |
|  |  | Simpson | 0.43 | 1 | 0.51 |
|  |  | Chao1 | 0.78 | 1 | 0.38 |
|  |  | ACE | 0.65 | 1 | 0.42 |
|  |  | Observed | 1.04 | 1 | 0.31 |
|  | T2 | Fisher | 0.56 | 1 | 0.46 |
|  |  | Shannon | 0.13 | 1 | 0.72 |
|  |  | Simpson | 0.36 | 1 | 0.55 |
|  |  | Chao1 | 1.29 | 1 | 0.26 |
|  |  | ACE | 0.36 | 1 | 0.55 |
|  |  | Observed | 0.56 | 1 | 0.46 |
|  | T3 | Fisher | 0.00 | 1 | 0.96 |
|  |  | Shannon | 0.42 | 1 | 0.52 |
|  |  | Simpson | 1.39 | 1 | 0.24 |
|  |  | Chao1 | 0.91 | 1 | 0.34 |
|  |  | ACE | 0.42 | 1 | 0.52 |
|  |  | Observed | 0.00 | 1 | 0.96 |
| LowHy vs other | T1 | Fisher | 0.46 | 1 | 0.50 |
|  |  | Shannon | 0.00 | 1 | 0.98 |
|  |  | Simpson | 0.24 | 1 | 0.62 |
|  |  | Chao1 | 1.08 | 1 | 0.30 |
|  |  | ACE | 0.46 | 1 | 0.50 |
|  |  | Observed | 0.46 | 1 | 0.50 |
|  | T2 | Fisher | 1.18 | 1 | 0.28 |
|  |  | Shannon | 0.22 | 1 | 0.64 |
|  |  | Simpson | 0.04 | 1 | 0.84 |
|  |  | Chao1 | 2.10 | 1 | 0.15 |
|  |  | ACE | 1.91 | 1 | 0.17 |
|  |  | Observed | 1.18 | 1 | 0.28 |
|  | T3 | Fisher | 2.25 | 1 | 0.13 |
|  |  | Shannon | 5.66 | 1 | 0.02* |
|  |  | Simpson | 6.75 | 1 | 0.01** |
|  |  | Chao1 | 3.13 | 1 | 0.08 |
|  |  | ACE | 2.71 | 1 | 0.10 |
|  |  | Observed | 2.25 | 1 | 0.13 |
