# Supplementary material for: Microbiota diversity and hygienic behavior in a honey bee breeding population: Insights into Varroa resistance
Source: PLoS One. 2026 Apr 9;21(4):e0346605. doi: 10.1371/journal.pone.0346605 (PMC13065041; doi:10.1371/journal.pone.0346605)
Supplement: S3 Table — The table reports the F-values and p-values for each fixed effect included in the model (AvePin, genetic line, sampling timepoint, and the interaction between AvePin and timepoint). (DOCX) [file pone.0346605.s003.docx]

**S3 Table. Results of linear mixed-effects models testing the association between alpha diversity indices and hygienic behavior group (AvePin) across all samples**. The table reports the F-values and *p*-values for each fixed effect included in the model (AvePin, genetic line, sampling timepoint, and the interaction between AvePin and timepoint).

| **Index** | **Effect** | **F-value** | ***P*-value** |
| --- | --- | --- | --- |
| Shannon | AvePin | 0.37 | 0.69 |
|  | Genetic line | 1.60 | 0.15 |
|  | Timepoint | 10.81 | 5.30E-05*** |
|  | AvePin:Timepoint | 1.85 | 0.13 |
| Fisher | AvePin | 0.25 | 0.78 |
|  | Genetic line | 0.42 | 0.89 |
|  | Timepoint | 6.92 | 1.50E-03*** |
|  | AvePin:Timepoint | 1.27 | 0.29 |
| Simpson | AvePin | 1.42 | 0.25 |
|  | Genetic line | 2.01 | 0.07 |
|  | Timepoint | 16.57 | 5.39E-07*** |
|  | AvePin:Timepoint | 2.81 | 0.03* |
| Chao1 | AvePin | 1.39 | 0.26 |
|  | Genetic line | 0.10 | 1.00 |
|  | Timepoint | 12.48 | 1.34E-05*** |
|  | AvePin:Timepoint | 2.92 | 0.02* |
| ACE | AvePin | 1.40 | 0.25 |
|  | Genetic line | 0.24 | 0.97 |
|  | Timepoint | 14.28 | 3.18E-06*** |
|  | AvePin:Timepoint | 2.10 | 0.09 |
| Observed | AvePin | 0.25 | 0.78 |
|  | Genetic line | 0.43 | 0.88 |
|  | Timepoint | 6.75 | 1.73E-03*** |
|  | AvePin:Timepoint | 1.31 | 0.27 |
