# Supplementary material for: Microbiota diversity and hygienic behavior in a honey bee breeding population: Insights into Varroa resistance
Source: PLoS One. 2026 Apr 9;21(4):e0346605. doi: 10.1371/journal.pone.0346605 (PMC13065041; doi:10.1371/journal.pone.0346605)
Supplement: S4 Table — The table reports specificity, sensitivity, and accuracy for each group (low, medium, and high) at different timepoints. (DOCX) [file pone.0346605.s004.docx]

**S4 Table.** **PLS-DA results based on the three-group AvePin classification and the dichotomous contrast variables.** The table reports specificity, sensitivity, and accuracy for each group (low, medium, and high) at different timepoints.

| **Group** | **Timepoint** | **Specificity** | **Sensitivity** | **Accuracy** |
| --- | --- | --- | --- | --- |
| LowHy | 1 | 0.868 | 0.05 | 0.644 |
| MedHy |  | 0.037 | 0.783 | 0.507 |
| HiHy |  | 1 | 0 | 0.904 |
| LowHy | 2 | 0.86 | 0.158 | 0.667 |
| MedHy |  | 0.32 | 0.727 | 0.58 |
| HiHy |  | 1 | 0 | 0.913 |
| LowHy | 3 | 0.97 | 0 | 0.696 |
| MedHy |  | 0.25 | 0.8 | 0.609 |
| HiHy |  | 0.977 | 0 | 0.913 |
| HiHy vs others | 1 | 0.985 | 0.143 | 0.904 |
|  | 2 | 0.977 | 0 | 0.86 |
|  | 3 | 0.977 | 0 | 0.913 |
| LowHy vs others | 1 | 0.906 | 0.1 | 0.685 |
|  | 2 | 0.76 | 0.421 | 0.667 |
|  | 3 | 0.879 | 0.154 | 0.674 |
